# Safety profile of Japanese encephalitis vaccines: a comprehensive analysis of vaccine adverse event reports from 1993 to 2025

**DOI:** 10.3389/fpubh.2025.1647485

**Published:** 2025-11-06

**Authors:** Fujun Liu, Qibo Ran, Huajin Zhang, Zhongyu Li, Jing Chen

**Affiliations:** 1 State Key Laboratory of Biotherapy Center, West China Hospital, Sichuan University, Chengdu, China; 2 Department of Ophthalmology, West China Hospital, Sichuan University, Chengdu, China; 3 Department of Neurosurgery and Neuromodulation Center, West China Hospital, Sichuan University, Chengdu, China

**Keywords:** encephalitis vaccines, VAERS, adverse events, safety, disproportionality analysis

## Abstract

**Objectives:**

Japanese encephalitis is a severe mosquito-borne disease requiring effective prevention and ongoing vaccine safety monitoring. This study aimed to analyze the characteristics, severity, and reporting trends of adverse events associated with Japanese encephalitis vaccines (JEV) in the U. S. Vaccine Adverse Event Reporting System (VAERS). We also investigated the correlation between Google search trends and adverse event reporting.

**Methods:**

Data were extracted from VAERS (1993–2025). Descriptive statistics, including demographics and temporal trends, were performed. Disproportionality analysis used the reporting odds ratio (ROR) with a 95% confidence interval (CI). Google Trends analysis covered 2004–2025 (worldwide, topic: “Japanese Encephalitis Vaccine”).

**Results:**

A total of 1,384 reports (6,596 vaccine-event pairs) were retrieved. IXIARO accounted for 3,452 pairs, JE-VAX 1368, J-VAX 698, and “unspecific Brand” 1,078. Serious reports totaled 284 (20.5%), and fatal reports 11 (0.80%). The 18–29 years group comprised 39.52% (*n* = 547) of reports, while the ≥60 years group constituted 3.4% (*n* = 47), with no fatal outcomes reported in this demographic. Key disproportionality signals (RORs) included nausea (*n* = 134, ROR = 1.32), dizziness (*n* = 132, ROR = 1.33), pruritus (*n* = 127, ROR = 2.33), and urticaria (*n* = 122, ROR = 2.89). Noteworthy brand-specific signals: IXIARO (loss of consciousness *n* = 32, ROR = 2.88; seizure *n* = 17, ROR = 2.97), JE-VAX (encephalitis *n* = 5, ROR = 15.69; angioneurotic oedema *n* = 4, ROR = 68.17), and J-VAX (laryngospasm *n* = 11, ROR = 185.73). Google search volume showed strong temporal correlation with VAERS reports (2004–2025).

**Conclusion:**

This study revealed the reporting patterns of JEV vaccine adverse events in the VAERS database, indicating that a considerable proportion of reports were serious events, and allergic reactions such as pruritus, rash, and urticaria were prominent. The observed overlap between spontaneous reporting and online search trends suggests public awareness and information dissemination influence reporting patterns. These findings underscore the need for continued JEV safety surveillance and further investigation.

## 1 Introduction

The Japanese encephalitis is a serious, mosquito-borne viral disease that presents a significant public health risk, particularly in many parts of Asia and the Western Pacific region (1, 2). Specifically, Japanese encephalitis is endemic in the Indian subcontinent (including India, Pakistan, and Bangladesh), Southeast Asia (especially the Indonesian archipelago, Vietnam, and Thailand), and spread to Papua New Guinea and the Torres Strait islands of northern Australia (1, 2). Caused by the Japanese encephalitis virus, which is transmitted primarily through the bite of infected Culex mosquitoes, Japanese encephalitis can lead to devastating neurological complications, long-term disability, and death, especially in children (3). Japanese encephalitis impacts approximately 69,000 people globally each year, with a mortality rate of 20 to 30% and an annual loss of 709,000 disability-adjusted life years (4, 5). This underscores the critical need for effective preventive strategies to control its spread and mitigate its impact on vulnerable populations.

Vaccination stands as the most effective method for preventing Japanese encephalitis and has been instrumental in significantly reducing disease incidence in endemic areas (6). Various Japanese encephalitis vaccines (JEV), such as inactivated, live-attenuated, and chimeric types, have been developed and are extensively utilized worldwide (7). Recommendations for Japanese encephalitis vaccinations vary depending on geographic location and individual risk factors, but mass vaccination campaigns and routine immunization programs have played a vital role in disease control efforts (7).

Despite the proven efficacy of JEV in preventing infection and disease, concerns regarding their safety profile have been raised. Reports of adverse events following immunization (AEFI) have, at times, contributed to public hesitation and impacted vaccination uptake in certain regions, such as informal settlements in Nairobi, Kenya (8). While pre-marketing clinical trials and post-marketing surveillance have provided valuable safety data, a comprehensive and long-term analysis of real-world adverse event reporting is crucial to further refine the understanding of JEV safety and address potential concerns (9). However, the existing studies are limited by their scope, time period, or the specific vaccine types included, leaving potential gaps in the current knowledge regarding the full spectrum of adverse events associated with different JEV brands and their reporting patterns over an extended period (10–12).

Safety concerns, even when rare, can erode public trust in vaccination programs. This may lead to decreased coverage rates and potentially contribute to a resurgence of Japanese encephalitis cases (13). Therefore, a thorough investigation into the reported adverse events associated with JEV is essential to maintain confidence in these vital preventive tools and ensure the continued success of Japanese encephalitis control initiatives. This study aims to perform a comprehensive analysis of adverse event reports related to JEV submitted to the U. S. Vaccine Adverse Event Reporting System (VAERS) from 1993 to 2025. This research aims to offer valuable insights into the safety profile of JEV in a large, real-world setting by analyzing reported adverse event characteristics, evaluating potential disproportionality signals for specific events and vaccine brands using methods like reporting odds ratio (ROR), and examining trends in reporting over time. Additionally, we examine the potential correlation between JEV-related Google searches and the trends in spontaneous adverse drug reaction reporting. This analysis will contribute to a better understanding of JEV safety, inform ongoing safety monitoring efforts, and support evidence-based decision-making by public health authorities and healthcare providers, ultimately helping to maintain high vaccination rates and protect populations at risk of Japanese encephalitis.

## 2 Materials and methods

### 2.1 VAERS database

The VAERS served as the data source for this study. VAERS, established in 1990 and jointly administered by the Centers for Disease Control and Prevention and the Food and Drug Administration, is a national passive surveillance system aimed at identifying potential safety signals and generating data on adverse events following vaccination (14). Due to the spontaneous reporting nature of the VAERS system, establishing causal links between vaccines and reported adverse events using VAERS data alone is generally not feasible (15).

VAERS collects voluntary reports from a variety of sources, including healthcare providers, vaccine manufacturers, vaccine recipients, and other source. Each report collects information pertaining to the vaccinated individual (such as age and sex), details of the administered vaccine, including concomitant vaccines, and the description of the adverse event experienced. Adverse events are systematically coded by trained personnel using the Medical Dictionary for Regulatory Activities (MedDRA), a clinically validated, internationally standardized terminology (16). In MedDRA, reported signs, symptoms, or diagnoses are assigned one or more preferred terms (PTs) within a system organ class (16). Reports submitted to VAERS are classified as “serious” if they meet one or more criteria defined by the US Code of Federal Regulations (17). These criteria encompass events leading to death, life-threatening illness, hospitalization or its extension, congenital anomalies, permanent disability, or requiring medical intervention to prevent these outcomes.

### 2.2 Data collection and study population

For this study, our analysis encompassed all documented safety signals associated with JEV from March 1993 to February 2025 in the VAERS database. Those JEV were classified four types (IXIARO, JE-VAX, J-VAX; NO Brand) in VAERS. These classifications are based on vaccine product names as recorded in VAERS and represent distinct vaccine types: IXIARO (a cell culture-derived, inactivated vaccine), JE-VAX (a mouse brain-derived, inactivated vaccine that was discontinued globally and replaced by IXIARO), and J-VAX (another mouse brain-derived, inactivated vaccine). The ‘NO Brand’ category includes VAERS reports where the specific JEV brand was not specified. Signals from this category warrant particular caution due to its inherent heterogeneity and potential for misclassification or incomplete reporting, which may reflect reporting artifacts rather than true vaccine-specific safety concerns. Therefore, RORs derived from the ‘NO Brand’ category should be interpreted with caution and are considered less reliable for specific signal detection. This classification scheme allows for the comprehensive analysis of all JEV-related adverse event reports within the VAERS database, including those from earlier, now discontinued, vaccine formulations. Adverse events were coded using preferred terms (PTs) from the MedDRA terminology. Vaccine manufacturers were identified using the Manufacturer of Vaccine variable. While JE-VAX and J-VAX have been discontinued, their inclusion in this study provides valuable historical safety profiles of JVE in the U. S. and allows for a comprehensive understanding of long-term post-marketing surveillance data. This historical context is relevant for identifying enduring safety signals, understanding evolving adverse event patterns, and informing regulatory considerations for future vaccine development.

### 2.3 Signal detection methods and statistical analysis

Disproportionality analysis was conducted using the ROR. By intentionally omitting inclusion criteria related to patient demographics, adverse event severity, or reporting sources, we aimed to maintain population-level representativeness in this pharmacovigilance assessment. The reported data were categorized and analyzed based on key characteristics, including vaccine type, age, sex, seriousness of the event, and year of reporting. The ROR was calculated based on a 2 × 2 contingency table comparing the reporting of a specific adverse event with a specific JEV versus all other vaccines in VAERS. The formula for ROR is: ROR = (a/b)/(c/d), where ‘a’ is the number of reports for the specific AE-vaccine pair, ‘b’ is the number of reports for the specific vaccine but not the AE, ‘c’ is the number of reports for the AE but not the specific vaccine, and ‘d’ is the number of reports for neither the AE nor the specific vaccine. A minimum of three reports for the specific AE-vaccine pair (*n* ≥ 3) was required for ROR calculation. Statistical significance was defined by a lower bound of the 95% confidence interval (95% CI) of the ROR being greater than 1 (17). A *p*-value of 0.05 or less was used to determine statistical significance. Due to the large number of comparisons in disproportionality analysis, there is a risk of false positive signals from multiple testing. While we report all statistically significant RORs, we acknowledge that a formal False Discovery Rate (FDR) correction was not applied, as disproportionality analysis is primarily hypothesis-generating. Signals, especially those with small report counts, warrant further investigation through more robust epidemiological studies. Importantly, ROR estimates from passive surveillance systems like VAERS are susceptible to various reporting biases, including underreporting, differential reporting by event type or vaccine, and stimulated reporting (e.g., increased reporting following media attention). These biases can inflate or deflate ROR values, meaning observed associations warrant cautious interpretation and do not necessarily indicate causation. Descriptive statistical analysis was performed with R Studio (version 4.4.2) to summarize the key characteristics of the reports between all JEV and different categories. Line graphs were utilized to visualize trends in reporting over time.

### 2.4 Google trends analysis

To investigate the potential relationship between public interest and the reporting of adverse events following vaccination, we conducted a Google Trends analysis. This ecological analysis explores patterns of public interest in relation to reported adverse events, rather than establishing causal links, which provide additional contextual insights into public perception trends. Google Trends^1^ is an online platform that provides data on the relative search volume of specific terms or topics across different regions and time periods. The relative search volume is presented on a scale from 0 to 100, where each data point represents the search interest for a given term or topic relative to the highest point in the selected time series, which is normalized to 100 (17). Analysis can be performed using either specific search terms or broader topics, with the latter offering wider coverage by encompassing a group of related terms that share the same concept. In this study, we utilized search topics to capture a comprehensive range of related queries. The time period for data collection was aligned with the period of adverse event reports from VAERS, specifically from 2004 to 2025. The geographical focus of the search was set to worldwide to correspond with the scope of the adverse event data. We selected “All categories” and “Web search” for the analysis settings. The specific search topic used in this analysis was “Japanese Encephalitis Vaccine.” Google Trends data were accessed on April 5, 2025. Descriptive analysis was conducted to examine the temporal changes in the relative search volume for the selected topic during the study period. These trends in online search queries were then compared with the annual number of adverse event reports obtained from VAERS to visually assess potential correlations.

### 2.5 Ethics statement

This study was based on publicly available de-identified data from VAERS and Google Trends; therefore, ethical approval and informed consent were not required.

## 3 Results

### 3.1 Descriptive analysis

**Figure 1:**
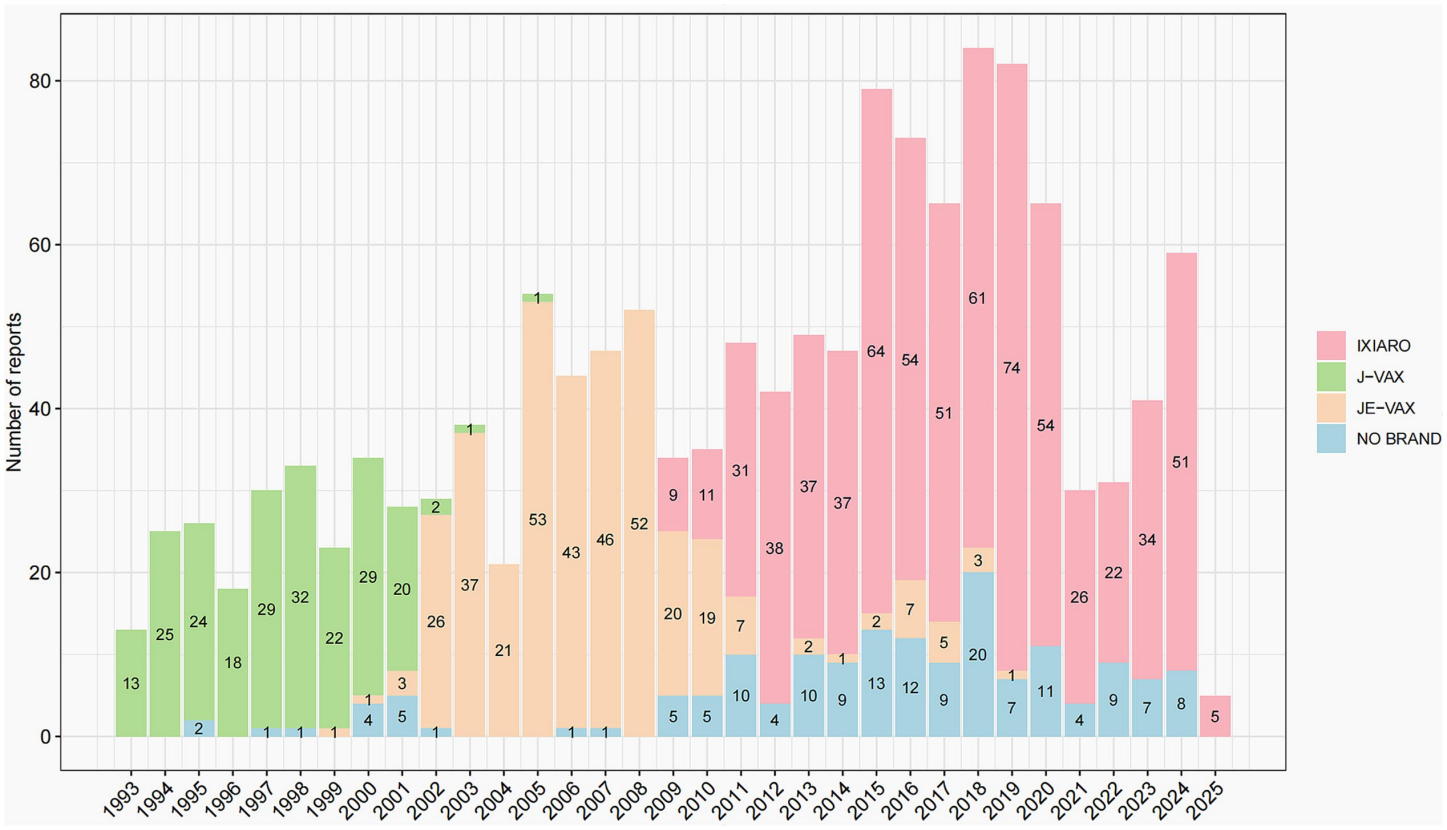
Annual distribution of JEV adverse event reports by brand, 1993–2025.

Over the 32-year period from 1993 to 2025, a total of 1,384 adverse event reports related to different JEV brands, corresponding to 6,596 vaccine-event pairs (3,452 IXIARO, 1368, JE-VAX, 698, J-VAX, 1078, NO Brand) were retrieved in VAERS, with Figure 1 illustrating the annual number of these reports. Reports associated with J-VAX were observed predominantly in the earlier years of the reporting period, with a peak of 32 reports in 1999 and a general decline thereafter, with sporadic reports in later years. The reports of JE-VAX were most prominent from 2003 to 2008, reaching a high of 53 reports in 2005, before decreasing significantly in subsequent years. Reports classified as “NO Brand” were present throughout the study period, with varying numbers, reaching a peak of 20 reports in 2018. However, the reports of IXIARO, show a distinct pattern, with a gradual increase starting around 2010 and a substantial rise from 2017 onwards, with annual report numbers consistently above 50 reports and reaching a peak of 74 reports in 2018. Overall, the temporal distribution of JEV vaccine adverse event reports in VAERS exhibit a shift in the dominant reporting brand over the study period, with IXIARO accounting for the majority of reports in recent years.

Table 1 presents the detail distribution of adverse event reports for JEV in the VAERS database (*N* = 1,384), of which 284 (20.5%) were classified as serious events, categorized by age, sex, along with the serious events and deaths. The highest proportion of reports originated from the 18–29 years age group, accounting for 39.52% (*n* = 547) of the total, followed by the 30–59 years age group at 34.68% (*n* = 480). Conversely, the <6 months and ≥60 years age groups contributed the lowest percentages of reports, at 0.43% (*n* = 6) and 3.4% (*n* = 47), respectively. Reports from females (52.9%) slightly outnumbered those from males (47.1%) across all age groups, excluding unknown sex. Serious events were distributed differently across age groups; the 18–29 years age group had the highest number of serious events (*n* = 41 in females, *n* = 55 in males), and the unknown age group also showed a substantial number of serious events (*n* = 22 in females, *n* = 31 in males). Fatal outcomes were reported in the 6 months–5 years (*n* = 1), 6–17 years (*n* = 3), 18–29 years (*n* = 1), 30–59 years (*n* = 1), and unknown age groups (*n* = 3).

**Table 1:** Characteristics of reports for Japanese encephalitis vaccines in VAERS.

Age group	Events reported	Sex	*N* (%)	Serious	Death
<6 months	6(0.43%)	F	4(0.29%)	1	0
		M	0(0%)	0	0
		U	2(0.14%)	0	0
6 months–5 years	72(5.2%)	F	34(2.46%)	14	0
		M	35(2.53%)	17	0
		U	3(0.22%)	2	1
6–17 years	84(6.07%)	F	49(3.54%)	9	1
		M	33(2.38%)	5	2
		U	2(0.14%)	0	0
18–29 years	547(39.52%)	F	258(18.64%)	41	1
		M	286(20.66%)	55	2
		U	3(0.22%)	1	0
30–59 years	480(34.68%)	F	212(15.32%)	22	0
		M	260(18.79%)	48	1
		U	8(0.58%)	0	0
≥60 years	47(3.4%)	F	29(2.1%)	7	0
		M	17(1.23%)	5	0
		U	1(0.07%)	0	0
Unknown	148(10.69%)	F	66(4.77%)	22	0
		M	62(4.48%)	31	3
		U	20(1.45%)	4	0
Total	1,384(100)			284(20.5%)	11(0.80%)

F, female; M, male; U, unknown.

### 3.2 Analysis of death reports

The fatal outcome rate of 0.80% (11 out of 1,384 reports) observed in the VAERS database represents the proportion of death reports among all adverse event reports for JEV (Table 1). This rate should not be confused with the mortality rate of Japanese encephalitis disease (typically 20–30% in symptomatic cases) (4, 5), as VAERS reports are submitted for events following vaccination. These deaths were distributed across several age groups: one death was reported in the 6 months–5 years age group, three deaths in the 6–17 years age group (two males, one female), three deaths in the 18–29 years age group (two males, one female), one death in the 30–59 years age group (male), and three deaths in the unknown age group (males). Notably, no deaths were reported in the <6 months and ≥60 years age groups. The 6–17 years, 18–29 years, and unknown age groups each reported three deaths. However, because the unknown age group has fewer reports (*n* = 148) than the 18–29 years group (*n* = 547), the death rate was higher in the unknown group (2.03%) than in the 18–29 years group (0.55%). The 6–17 years age group had the highest death rate (3.57%) among all groups.

The higher death rate in children and adolescents (6–17 years) might indicate an increased vulnerability in this age group, possibly due to immune factors or the nature of adverse events experienced. The relatively high number of deaths in the unknown age group may be due to underreporting of age information in severe cases, making this group worth further investigation. The relatively low death rate in the 18–29 years age group, despite accounting for the largest proportion of reports (39.52%) and serious events (*n* = 96), could suggest that young adults either experience less severe adverse events or have better access to care and recovery. However, these observations are based on a limited number of deaths and the inherent limitations of passive reporting systems like VAERS, and thus require cautious interpretation without implying causality. However, it is crucial to emphasize that these observations are based on a limited number of deaths and the inherent limitations of passive reporting systems like VAERS. Without access to detailed clinical narratives, or complete medical histories—which are typically unavailable in the public VAERS data used here—it is impossible to determine whether the vaccination played a causal role in these fatalities. Therefore, these findings should be interpreted with caution and do not imply causation.

### 3.3 Disproportionality analysis

This analysis was conducted on 6,596 vaccine-event pairs linked to JEV. We compared these JEV with other vaccines in the database. Table 2 lists the top 20 reported adverse events associated with JEV that showed a statistically significant disproportionality signal in the VAERS database. The top five most frequently reported events with positive signals were nausea (*n* = 134, ROR = 1.32, 95%CI: 1.11–1.57), dizziness (*n* = 132, ROR = 1.33, 95%CI: 1.12–1.58), pruritus (*n* = 127, ROR = 2.33, 95%CI: 1.95–2.77), rash (*n* = 126, ROR = 1.81, 95%CI: 1.51–2.16), and urticaria (*n* = 122, ROR = 2.89, 95%CI: 2.42–3.46). Among the listed events, urticaria showed the highest ROR (2.89), followed by pruritus (2.33), suggesting stronger disproportionality signals for these allergic reactions. Other reported events with statistically significant RORs included vomiting (*n* = 95, ROR = 1.62), paraesthesia (*n* = 79, ROR = 2.03), syncope (*n* = 63, ROR = 1.81), and less frequent but potentially more serious events like loss of consciousness (*n* = 39, ROR = 1.83) and seizure (*n* = 28, ROR = 2.56). The findings underscore a spectrum of adverse events linked to JEV in VAERS, exhibiting different frequencies and signal strengths.

**Table 2:** Most-reported positive adverse drug reactions for JEV.

PT	*N*	ROR (95%Cl)
Nausea	134	1.32(1.11–1.57)
Dizziness	132	1.33(1.12–1.58)
Pruritus	127	2.33(1.95–2.77)
Rash	126	1.81(1.51–2.16)
Urticaria	122	2.89(2.42–3.46)
Vomiting	95	1.62(1.32–1.98)
Paraesthesia	79	2.03(1.62–2.53)
Erythema	77	1.4(1.12–1.75)
Syncope	63	1.81(1.42–2.33)
Hyperhidrosis	54	1.88(1.44–2.46)
Loss of consciousness	39	1.83(1.33–2.51)
Abdominal pain	33	2.02(1.43–2.84)
Influenza like illness	31	1.57(1.1–2.23)
Pallor	30	1.94(1.35–2.77)
Rash pruritic	30	2.59(1.81–3.7)
Seizure	28	2.56(1.76–3.71)
Hypersensitivity	25	2.02(1.36–2.99)
Oedema peripheral	25	1.96(1.32–2.9)
Rash erythematous	25	1.62(1.09–2.4)
Musculoskeletal stiffness	23	1.78(1.18–2.68)

PT, preferred term; N, number; ROR, reporting odds ratio; CI, confidence interval.

We then analyzed each JEV in comparison with the other JEV. Table 3 presented the 20 most frequently reported adverse events with statistically significant disproportionality signals for each JEV brand (IXIARO, JE-VAX, J-VAX, and NO Brand) in the VAERS database, along with their report counts (N) and RORs with 95% CI. The most reported JEV was IXIARO, which resulted in statistical significance in 135 out of 1,050 vaccine reaction pairs (12.86%). Table 3 presents IXIARO’s data, highlighting the top 20 statistically significant AEFIs, most of which are already documented in the comprehensive JEV characteristics summary. For IXIARO, commonly reported events with positive signals included dizziness (*n* = 77, ROR = 1.48), rash (*n* = 51, ROR = 1.39), vomiting (*n* = 43, ROR = 1.4), and paraesthesia (*n* = 41, ROR = 2.01). Notably, IXIARO also showed a strong signal for immediate post-injection reaction (*n* = 25, ROR = 4.67) and potentially serious events such as loss of consciousness (*n* = 32, ROR = 2.88) and seizure (*n* = 17, ROR = 2.97).

**Table 3:** Most-reported adverse drug reactions for each JEV and corresponding RORs.

Vaccine	Adverse event	*N*	ROR (95% CI)
IXIARO	Dizziness	77	1.48(1.18–1.86)
	Rash	51	1.39(1.05–1.83)
	Vomiting	43	1.4(1.03–1.89)
	Paraesthesia	41	2.01(1.48–2.74)
	Urticaria	40	1.8(1.32–2.45)
	Syncope	36	1.98(1.43–2.75)
	Loss of consciousness	32	2.88(2.03–4.07)
	Hyperhidrosis	31	2.06(1.45–2.94)
	Post-injection reaction	25	4.67(3.15–6.92)
	Influenza like illness	20	1.94(1.25–3)
	Pallor	19	2.34(1.49–3.68)
	Migraine	18	2.66(1.68–4.23)
	Seizure	17	2.97(1.84–4.78)
	Rash Pruritic	16	2.63(1.61–4.3)
	Rash erythematous	14	1.73(1.03–2.93)
	Musculoskeletal stiffness	13	1.92(1.12–3.32)
	Presyncope	11	2.97(1.64–5.37)
	Skin lesion	11	7.01(3.87–12.67)
	Rash papular	10	3.47(1.86–6.45)
	Flushing	10	2.07(1.11–3.85)
J-VAX	Pruritus	40	7.2(5.23–9.9)
	Pyrexia	35	1.67(1.19–2.34)
	Headache	29	1.56(1.07–2.26)
	Rash	28	3.88(2.66–5.66)
	Urticaria	26	5.93(4.01–8.77)
	Nausea	22	2.07(1.35–3.17)
	Dizziness	19	1.82(1.15–2.87)
	Myalgia	19	2.31(1.47–3.65)
	Hypersensitivity	18	16.43(10.29–26.25)
	Vasodilatation	17	25.94(16.02–41.99)
	Paraesthesia	15	3.67(2.2–6.13)
	Dyspnoea	14	1.88(1.11–3.19)
	Asthenia	14	2.47(1.45–4.19)
	Syncope	13	3.57(2.06–6.18)
	Vomiting	13	2.1(1.22–3.64)
	Laryngospasm	11	185.73(102.03–338.0)
	Diarrhea	10	2.27(1.22–4.24)
	Hyperhidrosis	10	3.31(1.77–6.18)
	Chest pain	10	2.5(1.34–4.66)
	Oedema peripheral	10	7.48(4.01–13.97)
JE-VAX	Headache	50	1.36(1.03–1.81)
	Dizziness	31	1.51(1.06–2.15)
	Erythema	28	2.48(1.7–3.6)
	Dyspnoea	23	1.57(1.04–2.37)
	Chest discomfort	13	2.92(1.69–5.04)
	Abdominal Pain	12	3.55(2.01–6.26)
	Flushing	9	4.72(2.45–9.09)
	Dysphagia	8	5.44(2.71–10.9)
	Convulsion	5	2.95(1.23–7.1)
	Encephalitis	5	15.69(6.51–37.79)
	Angioneurotic oedema	4	68.17(25.45–182.63)
	Balance disorder	4	2.71(1.01–7.22)
	Depressed of consciousness	4	6.53(2.45–17.44)
	Anorexia	3	5.84(1.88–18.13)
	Coordination abnormal	3	9.79(3.15–30.42)
	Dermatitis exfoliative	3	105.22(33.65–328.99)
	Eyelid oedema	3	12.69(4.08–39.44)
	Face oedema	3	5.02(1.62–15.59)
	Facial palsy	3	16.15(5.2–50.2)
	Hearing impaired	3	89.97(28.8–281.02)
NO brand	Pyrexia	47	1.44(1.08–1.93)
	Seizure	11	6.18(3.41–11.19)
	Febrile convulsion	6	9.05(4.05–20.19)
	Upper respiratory infection	6	22.4(10.03–50.02)
	Vaccination site swelling	6	4.54(2.04–10.14)
	Vaccination site erythema	5	3.99(1.66–9.62)
	Angioedema	5	9.22(3.83–22.22)
	Cellulitis	5	3.05(1.27–7.35)
	Hypersensitivity	5	2.47(1.03–5.95)
	Pneumonia	4	2.75(1.03–7.33)
	Irritability	4	3.37(1.26–9)
	Encephalitis	3	11.93(3.84–37.06)
	Bronchitis	3	8.84(2.84–27.46)
	Viral Infection	3	5.06(1.63–15.71)
	Arthritis	3	4.37(1.41–13.56)
	Vaccination site induration	3	11.9(3.83–36.98)
	Sepsis	3	5.95(1.92–18.48)
	Skin infection	3	61.34(19.69–191.1)
	Henoch–Schonlein purpura	3	33.36(10.72–103.8)
	Encephalopathy	3	15.33(4.93–47.63)

*N*, number; ROR, reporting odds ratio; CI, confidence interval.

Compared to other JEV in VAERS, JE-VAX demonstrated statistical significance in 58 out of 387 vaccine-reaction pairs. The top 20 most reported PT of JE-VAX was list (Table 3). JE-VAX reports highlighted signals for headache (*n* = 50, ROR = 1.36), dizziness (*n* = 31, ROR = 1.51), and erythema (*n* = 28, ROR = 2.48). Of note, the disproportionality analysis detected strong signals for JE-VAX and more severe events, including encephalitis (*n* = 5, ROR = 15.69) and angioneurotic oedema (*n* = 4, ROR = 68.17). While these high RORs warrant attention due to the potential severity of the events, it is crucial to note that they represent signals from passive surveillance necessitating further investigation and do not, by themselves, establish causation.

For J-VAX, statistically significant disproportionalities were observed in 46 out of 166 vaccine-reaction pairs. Table 3 shows the 20 more reported AEFIs for J-VAX. J-VAX showed strong signals for pruritus (*n* = 40, ROR = 7.2), rash (*n* = 28, ROR = 3.88), and urticaria (*n* = 26, ROR = 5.93), indicating a propensity for allergic reactions. Furthermore, J-VAX had extremely high RORs for injection site hypersensitivity (*n* = 18, ROR = 16.43) and laryngospasm (*n* = 11, ROR = 185.73), although the number of reports for laryngospasm was relatively low. The extremely high ROR for laryngospasm, while statistically significant, is based on a small number of reports and may be influenced by reporting artifacts or biases. As with all signals from disproportionality analysis, this finding is exploratory and requires confirmation.

Analyses of the ‘NO Brand’ category should be interpreted with the understanding that this group is inherently less precise, and signals identified within it may be confounded. Disproportionalities for NO Brand were statistically significant in 51 out of 553 vaccine-reaction pairs. The reports classified as “NO Brand,” pyrexia was the most frequent event with a positive signal (*n* = 47, ROR = 1.44). Table 3 presented the top 20 more reported AEFIs for NO Brand. This category also exhibited strong signals for seizure (*n* = 11, ROR = 6.18), febrile convulsion (*n* = 6, ROR = 9.05), and upper respiratory infection (*n* = 6, ROR = 22.4). Notably, skin infection showed a very high ROR (*n* = 3, ROR = 61.34 [95% CI, 19.69–191.1]); however, the small number of cases (*n* = 3) and wide confidence interval (19.69–191.1) urge caution in interpretation, as such disproportionate reporting might be influenced by coding artifacts or reporting bias rather than a true safety signal. Therefore, RORs for the ‘NO Brand’ category should be regarded with particular skepticism due to the heterogeneity and potential misclassification within this group, rendering them less reliable for specific signal detection compared to branded vaccines. In summary, these brand-specific analyses reveal distinct adverse event profiles and varying signal strengths across different JEV products.

### 3.4 Google trends analysis

**Figure 2:**
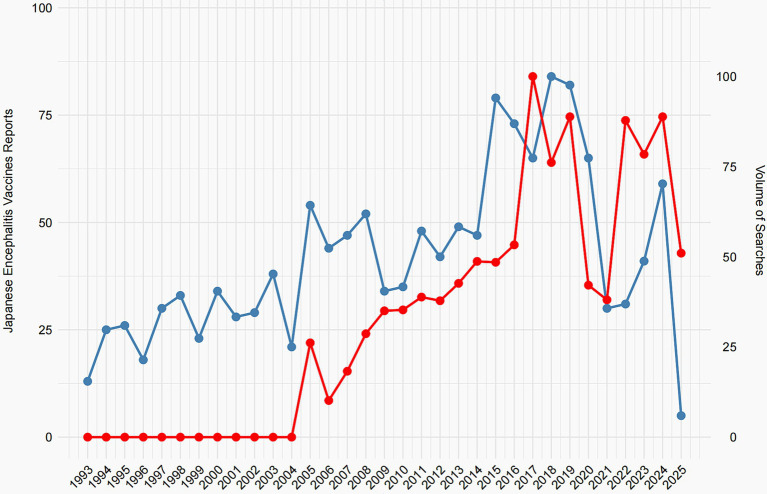
Annual trends in JEV adverse event reports and Google Search volume, 1993–2025.

Figure 2 compares the annual number of JEV-related adverse event reports in VAERS (blue line, left *y*-axis) with the relative search volume for JEV on Google Trends (red line, right *y*-axis) from 1993 to 2025. Google search data are only available from 2004 onward (Google’s founding year); hence, values before 2004 are zero. The top related queries retrieved by Google Trends included terms related to JEV side effects, indicating public interest in vaccine safety. The VAERS reports show fluctuating numbers, generally staying below 60 reports per year before 2017, followed by a sharp rise peaking at over 75 reports in 2017–2018, a decline, and another increase in 2023–2024. The Google search volume for JEV displays a general upward trajectory after 2004. Notably, the temporal patterns of the two data streams show increasing alignment after 2004, with similar peaks and troughs (e.g., the rise around 2017–2018 and 2023–2024).

The primary significance of this analysis lies in exploring the potential influence of public awareness and media attention—proxied here by search volume—on spontaneous adverse event reporting. The observed co-movement suggests that heightened public interest, possibly driven by news or online discussions about JEV safety, may coincide with increased reporting to VAERS. This underscores a key limitation of passive surveillance systems: reporting rates can be influenced by external factors unrelated to the actual safety profile of the vaccine. Consequently, spikes in reports should not be interpreted as evidence of increased risk without further investigation. Future studies incorporating media event data could help elucidate the drivers behind these correlations. Data for 2025 are incomplete as of the analysis date (February 1, 2025).

## 4 Discussion

This study provides a descriptive overview of adverse event reports related to JEV submitted to the VAERS in the United States from 1993 to 2025. Our analysis of temporal trends, reporter demographics, and disproportionality (using ROR methods) provides updated insights into the long-term safety profile of all JEV and their specific brands.

Our descriptive analysis revealed a clear temporal pattern in the distribution of reported adverse events, reflecting the evolution of JEV usage in the US. Reports associated with J-VAX were predominant in the 1990s, followed by JE-VAX in the mid-2000s, before IXIARO became the dominant vaccine from the 2010s onwards. The observed decline in reports for older brands (J-VAX and JE-VAX) and the concurrent increase in reports for IXIARO after its licensure closely align with the documented introduction and wider adoption of newer vaccine formulations (18). This pattern demonstrates the critical influence of vaccine utilization patterns on reporting volume in passive surveillance systems. Such analysis is essential to avoid misinterpretation of temporal trends, such as attributing an increase in overall reports to a safety issue when it may primarily reflect increased use of a newer vaccine. The demographic analysis indicated that individuals aged 18–29 years and 30–59 years accounted for the majority of reports, which may reflect targeted vaccination campaigns or increased travel to endemic areas among these age groups (18, 19). The slight female preponderance in reporting aligns with observations in other vaccine safety studies utilizing passive surveillance systems, which could be attributed to differences in healthcare-seeking behavior or reporting tendencies (14, 20). While serious events were reported across various age groups, the substantial number of serious reports in the unknown age group warrants attention and highlights a limitation of passive surveillance data where complete demographic information may be missing (21). The descriptive analysis of fatal reports, although representing a small proportion of total reports, highlights the rare fatal outcomes reported following JEV. Due to the inherent limitations of passive surveillance systems like VAERS—where reports vary in detail and completeness and do not inherently establish causation—careful evaluation of individual cases, including review of medical records and autopsy reports where available, is essential. This process, typically conducted by regulatory agencies, may help distinguish potential safety signals from coincidental events.

The disproportionality analysis revealed statistically significant signals for various adverse events linked to JEV in the VAERS database. Common systemic reactions, including nausea, dizziness, rash, and urticaria, exhibited positive RORs, indicating they were more frequently reported after JEV than other vaccines in VAERS. These findings are generally consistent with the known reactogenicity profiles of various vaccines (22). More specific to allergic reactions, pruritus and urticaria exhibited higher RORs, which could be related to vaccine components or individual predispositions (23). Regarding potential mechanisms underlying the observed adverse events, the prevalence of reactions such as urticaria and angioedema might suggest involvement of mast cell degranulation. Furthermore, as noted by Duggan and Plosker in their comparison of IXIARO with an aluminum hydroxide adjuvant placebo, remarkably similar adverse reaction rates were observed, indicating that some reactions could be attributed to vaccine excipients (e.g., aluminum adjuvant) rather than the viral antigen itself, or even to the vaccination process independent of specific vaccine components (24). This highlights the complex interplay of vaccine components and individual biological responses in mediating adverse events.

In our study, IXIARO, the currently dominant vaccine in US reports, showed signals for expected reactions like dizziness and vomiting, but also for potentially more serious events such as syncope, loss of consciousness, and seizure, albeit with lower RORs than some events associated with older brands. Research by Ingrid et al. found that the overall reporting rate for IXIARO was 15.2 cases per 100,000 doses, with serious adverse events accounting for 1.8 cases, and no deaths reported (10). Among these surveillance signals, strong associations were observed specifically for JE-VAX regarding more severe events, including encephalitis (5 reports, ROR = 15.69, 95% CI, 6.50–37.89) and angioneurotic oedema (4 reports, ROR = 68.17, 95% CI, 23.36–198.81). Notably, whereas angioedema is listed as a potential adverse reaction in the U. S. Prescribing Information for JE-VAX, encephalitis is not explicitly labeled for any current JEVs, including IXIARO. This discrepancy, combined with the limited number of reports, underscores VAERS’s ability to detect potential safety signals that may not yet be fully characterized in product labels. These findings warrant further investigation through more robust study designs (10). Similarly, J-VAX showed strong signals for allergic reactions and remarkably high RORs for events like laryngospasm and injection site hypersensitivity. The allergic reaction signals for the J-VAX vaccine may be related to its formulation, which aligns with other studies indicating that older vaccine formulations may lead to a higher risk of allergic reactions (22, 25). For laryngospasm, while showing a high ROR, the absolute number of reports remains low. A review of existing regulatory labels (e.g., U. S. Prescribing Information, EMA/FDA regulatory reports) for current JEVs does not explicitly list laryngospasm as a labeled adverse reaction. This necessitates a careful case review by regulatory agencies to assess whether these represent novel signals or miscoding. Signals for events like encephalitis warrant particular attention, and their consistency with findings from other pharmacovigilance databases or active surveillance would strengthen their significance.

Our findings broadly align with existing literature on JEVs safety. For instance, the frequencies of commonly reported adverse events such as headache and fatigue are largely consistent with those reported in clinical trials and meta-analyses, such as Kling et al.’s systematic review (2020). However, discrepancies for rare but serious adverse events, such as the disproportionate reporting of encephalitis observed in our VAERS analysis, warrant further attention and highlight the complementary role of real-world pharmacovigilance data in identifying signals that may not be fully captured in controlled clinical trial settings. While some reactions like urticaria are well-documented, others, especially those with high RORs but low absolute numbers, require confirmation through active surveillance systems or further targeted studies.

Our analysis of Google Trends revealed an interesting correlation between public search volume for JEV and the number of adverse event reports in VAERS, particularly in recent years. This observed co-movement suggests that increased public interest and online information seeking regarding JEV may be associated with a rise in reported adverse events. While this finding does not establish causality, it highlights the potential influence of public awareness, media coverage, and online activity on passive surveillance data (26). This underscores the importance of clear and accurate public health communication regarding vaccine safety to manage public perception and mitigate potential reporting biases.

This study’s strengths lie in its use of an extensive, long-term VAERS dataset, offering an extensive overview of JEV adverse event reporting in a real-world context over three decades. The comprehensive analysis covering descriptive statistics, death reports, and disproportionality analysis, both for pooled data and individual brands, offers a detailed examination of reported safety signals. The use of ROR is a well-established method for signal detection in passive surveillance systems. However, this study is subject to several limitations inherent to the VAERS passive surveillance system. Reports submitted to VAERS are voluntary and may be incomplete, lack detailed clinical information, or be subject to reporting biases (e.g., overreporting of certain events or underreporting of others). Importantly, a significant limitation of this study is the reliance on publicly available VAERS data, which, by its nature, lacks the full case narratives and detailed medical records that are typically available in the underlying, non-public reports. This absence of some clinical information significantly hinders our ability to clinically validate reports, ascertain causality, or conduct an in-depth review of potential serious associations, particularly for rare events or fatal outcomes. The inability to calculate incidence rates stems from the unknown total number of vaccinated individuals, complicating the assessment of the true frequency of adverse events. VAERS data alone cannot establish causality; a temporal link between vaccination and an adverse event does not imply the vaccine caused it. The signals detected through disproportionality analysis are hypothesis-generating and require confirmation through more rigorous epidemiological studies with active surveillance or comparative designs. The “NO Brand” category highlights the issue of missing vaccine information, which can limit the specificity of safety assessments. Finally, our study focused solely on the U. S. VAERS database. Future research could integrate data from other national passive surveillance systems (e.g., EudraVigilance in Europe, Canada’s CAEFISS) or active surveillance datasets to assess the consistency of the signals identified here.

Given the limitations of passive surveillance, future research should aim to confirm these signals through more robust study designs, such as active surveillance systems (e.g., CDC’s V-safe, which actively monitors vaccinated individuals), nested case–control studies leveraging large healthcare databases, or retrospective cohort studies. Such approaches would allow for better control of confounding factors and potentially establish causal relationships.

## 5 Conclusion

This study presents a comprehensive analysis of JEV adverse event reports within the US VAERS from 1993 to 2025. Our findings reveal a dynamic reporting landscape reflecting changes in vaccine brand utilization over time, with IXIARO accounting for the majority of recent reports. Through disproportionality analysis, we identified signals for common systemic reactions as well as potentially more serious adverse events, with varying signal strengths observed across different vaccine brands, notably for older formulations like JE-VAX and J-VAX. Of particular interest is the observed correlation between Google search trends and VAERS reports, suggesting a potential influence of public interest on reporting patterns. Despite the inherent limitations of passive surveillance data, this study contributes valuable real-world evidence to the ongoing safety monitoring of JEV. Continued active surveillance, comparative effectiveness and safety studies, and in-depth investigations into the potential mechanisms underlying detected signals are essential for further refining the understanding of JEV safety and ensuring informed decision-making regarding vaccination recommendations.

## Data Availability

The raw data supporting the conclusions of this article will be made available by the authors, without undue reservation.

## Footnotes

^1^ https://trends.google.com/trends
